# Pericementitis

**Published:** 1882-07

**Authors:** G. A. Mills


AMERICAN JOURNAL

OF

DENTAL SCIENCE.

Vol. XVI. THIRD SERIES—JULY, 1882. No. 3.

### ARTICLE I.

### *Pericementitis.*

### ITS MANIFESTATIONS IN THE ORAL CAVITY, AND ITS SERIOUS EFFECTS UPON THE GENERAL HEALTH.

BY G. A. MILLS, D. D. S.

[From Proceedings of the Medical Society of the County of Kings, N. Y.]

Pericementitis, its manifestations in the oral cavity and the serious effects upon the general health, is considered by me of great import to the public whom we are called upon to serve, and certainly should be so considered by us as guardians of the public health. This is the purpose I have in appearing before your body to-night, that I may present to your intelligence, facts that will awaken your co-operative interest by taking cognizance of the prevalence of this destructive disorder.

While I fully recognize the fact that the department of the healing art, of which it is my pride to be a member, is a specialty of the general body, yet I am fully aware of another fact—as you doubtless are—that the attention of the oral cavity, comprising the teeth and their allied structures, has been, in the eariler times, so unnoticed that it created a necessity in the animal economy for the branch or specialty which I represent; and yet while we have the recognition of an unparalled progress, strange it may seem when I acquaint you with the incontrovertible truth that we have not been able to meet or stay the tide of this cyclone of destructiveness, as yet, to any considerable extent, while the part that has called into action the mechanical ability, has matured into a high degree of excellence. And now the department of education that presides over the culture of surgical ability is being recognized as *the* important factor and requisite to cope with those of a larger range of cultured ability. So fast as this shall occur in individual or aggregate cases, there will be but one mind regarding the appropriate recognition of fraternal counsel.

The subject which I come to speak to you of is not a new one; it may be, for aught I know, as old as disease itself. It has not been an unnoticed one either, in the general literature of the healing art; it has been characterized under a variety of nomenclature, with which you are more or less familar. So far as my understanding has reached, it has been generally considered as the result of advanced age, and doubtless, for this reason, that the expressions have been the more generally noticed because of the advanced condition so prevalent at this stage of life; while, on the other hand, with the light we now have, it is clearly shown that the mass of cases have their beginnings in early life, and because of the untrained perception, the minor expressions have failed to gain the notice they now prove to be worthy of. Bleeding and sponginess of the gums and the accumulation of lime, so-called tartar, have been lightly taken into account, and therefore as lightly dealt with, being met simply with some feeble astringent, or by the semi-barbarous operation of scraping or scaling the teeth—of which some of you may still retain a vivid recollection. This being slightly considered, it was found necessary to repeat this inquisition with frequency, and with little, if any benefit, beyond the comfort of the external surroundings of the teeth and gums; but further on, as a larger indentification of complications with personal discomforts associated, the interrogation has been not uncommonly put: "Why this state of affairs? And is there no remedy?" And as often received the almost stereotyped answer: "It is not amenable to successful treatment," while untold suffering has accrued, and the loss of thousands upon thousands of teeth that were yet untouched by the disintegrating process of caries. We have often, also, after our best and perfected operations by fillings, been obliged to acknowledge that these efforts were not enough to prove the highest efficiency of our calling; for, did it profit anything to be able to save the tooth, and, after all, the socket be swept away by disease? Fortunately, out of every great emergency there always seems to be provided some Moses to lead into a larger freedom.

This brings me to a point in this paper which will go into history. I now call your attention to facts that you may not be particularly familiar with, and which will serve to answer the question why this subject has come so persistently and hopefully to be considered during the few later years. I hold this particular feature of this paper to be so pertinent and just, that I cannot withhold its introduction. To Dr. John M. Riggs, of Hartford, Conn., is due the credit of the revival of interest in this subject; a gentleman of no ordinary culture, both of A. B. and M. D., and one who has no peer, in my estimation, in his State, as a true professional man. He has been for some forty years an earnest investigators of the practical details of office practice. His characteristics being somewhat peculiar to himself, he did not come to the arena of discussion in our calling so readily as others, and therefore we did not get the benefit of his observations in this direction so soon as we might otherwise have done. And yet it may be that he came in due time. It may not detract anything of interest to state that Dr. Riggs was the gentleman associated with Dr. Wells, of Hartford, in his experiments with nitrous oxide gas, Dr. Riggs extracting from Dr. Well's mouth a tooth, it being the first operation of this nature under the effect of an anæsthetic ever made. To use the words of the doctor himself, he says: "This disturbance, under consideration, early enlisted his attention," and he came to know that he was meeting with an increasing success beyond that, which he discovered, of his fellow practitioners, and did occasionally drop into limited conversation regarding it, so that it came to be known that he was pursuing a line of treatment not general, if at all known of, by the profession of dentists.

Dr. Riggs' public announcement of his views created no little credulity and curiosity, they being entirely new and different from anything then incorporated in the general literature. He claims to have found it necessary not only to remove the external deposits about the necks of the teeth, but at a certain stage of the disease to follow on to the margins of the process and trim away that portion that had become a foreign body by inflammatory action, instancing it as a principal recognized in general surgery (*i. e.*,) to cut back to the life line or into it, and thus establish a healthy action.

I will say, in passing, that human nature is quite the same in our department as in others, and the matter was quietly waived aside by some and vigorously attacked by others—I refer to the older members. Counter claims for originality were put in, but time has not evidenced the truth of them; and I do not hesitate to say that the truth of Dr. Riggs' claim had been fully established to the minds of all fair-minded men who have been cognizant of the discussions that have taken place.

The outcome of this has been the origin of the nomenclature "Riggs' Disease," which has become so familiar among us. Dr. Riggs' has, as a result of his attention to this subject, devised a set of instruments for treating this disease, so constructed as to be fully able to search out the disturbing points, thus producing results which warrant the saying that it is a surgical operation of no mean order, and one that cannot be familiarized without extreme care and intelligent training. A novice can do much harm and afflict his patient severely, while the trained hand, presided over by an intelligent mind, can become an alleviator of great suffering and bring much physical harmony out of decided unhealth.

This subject did not gain the attention of our national body until the session of 1877, held in Chicago. In 1876 and 1877 I published a series of six articles in the *Cosmos*, under the title of "What I know about Riggs' Disease." These articles have been very extensively circulated and favorably commented upon in this country and foreign ones. Following these articles, Dr. Rehwinkle, of Chillicothe, Ohio, an able writer, presented an article to the national body entitled Pyorrhea Alveolaris, meaning the pus-discharging sockets, and has also been defined as catarrh of the gums. Before this body the subject was largely and ably discussed by many of our best men, and since that time it has received more or less attention throughout the societies of our specialty, and, as you are aware, occupied a place among the subjects at the International Congress held at London. There it was brought forward under the title of "Premature Wasting of the Alveolar Process." Dr. Riggs was present and engaged in the debate. During the last year the subject was taken up by the Odontological Society of New York City, being introduced by a paper, the product of Dr. Niles, of Boston, a graduate of the Harvard Dental Department, and was christened under the title "The Calcic and Phosphatic Diathesis of Odontolithus." Extensive discussions followed the paper, and both the discussions and the paper may be found in the published proceedings of the society. I have felt that I could not be faithful to this subject without giving some idea of the difficulty and opposition that has encountered a matter of such vast interest and importance, but the cheering thought brings encouragement to those who have labored devotedly to place this matter in its proper position and to enlarge its sphere of usefulness in the alleviation of human suffering.

### PERICEMENTUM PERICEMENTITIS AND ITS HISTOLOGICAL FEATURES.

To Dr. C. W. F. Boedecker, of New York City, we are indebted for the ablest paper upon this subject yet furnished us. It is the product of faithful observation made by actual work with the microscope in the laboratory of Carl Heitzman, and furnishes us an understanding of this subject that makes the way quite plain. These articles can be found in the *Cosmos*, and will be profitable reading to any who may feel an interest in the matter.

As it is not my purpose to environ this paper with any extended details upon the scientific aspect, I will not enter largely upon its histological features, only noting the fact that they are comprised within both the myoxomatous and fibrous connective tissue series, the former in the early life and the latter more advanced. This will account for the variable associations of discomforts between the early and later disturbances, the former attended with a less degree of pain ; and yet, under favorable circumstances, a greater rapidity of progress is made, for the reason that the one is endowed with less resistance than the other

Pericementum has a connection of continuity both with myxomatous, or gum tissue, and the periosteum. By being so allied to the cementum, a continued disturbance of the periosteum results in the complication of the disease, and the destructiveness not only of these but also the osseous portions forming the socket.

Pericementitis is an expression of a greater or less degree of debility resultant upon nerve degenercy. It has a variety of phases, yet is more generally manifested at the peripheral margin of the gum. This is characterized sometimes by slight tinge of congestion, changing the appearance from a normal, pinkish color to one of deeper red or purple, and at others to an anæmic or bloodless, or colorless appearance. This is followed by a detaching, or relaxing the constriction of the membrane about the neck of the tooth. This is followed by the appearance of foreign substances, or by the absence of them, and an extended detachment of the tissues about the whole or part of the neck of the tooth. The character of the inflammation—be it acute or otherwise, destructive or less so—is determined by the constitutional powers of resistance or the opposite. By this, I mean if there is a quantity of power to aid in the producing of the *quantum sufficit* of such equality of proportions to establish a normal status of repair; or if it be equal in one and deficient in another, these must necessarily be so overpowered as to result in an overplus of disturbances of territory, the weak succumbing to the strong. This may be the awakening of the bond or bonds of energy in a normal degree, but yet being met by a bond of enfeebled affinit the results can only be destructive in a greater or less degree. Hence the necessity of something in the supply of nutriment that will be adapted to restore the enfeebled bond to its normal power of requisite affinity, so that the equality of waste and repair may be normally adjusted. This statement may or may not seem somewhat obscure, but my belief is that when the *modus operandi* of inflammatory action is understood, it will then be made possible to accept this. The time has, in a large measure, arrived for the physician of the future to establish the fact that his mission is of prevention rather than cure. We, in our investigations, look for exciting causes. To say that I am fully prepared to answer at this point would possibly seem quite like assumption. Many views and theories are advanced. Some attribute the cause to the presence of deposits of lime and their mixtures (so-called tartar.) Some claim that these are the results of inflammatory action, and still others call them sanguinary deposits, or residuum of the broken down tissues, blood, etc. Now I cannot accept any of these views as the *cause* of the disease. The *cause* is *in esse.* By this, I may mean, or will so express as a general term, nerve degeneracy. This gives rise to the question of definition for that. These questions all give rise to the acknowledgment of the impossibility of meeting all the points except upon the knowledge of the organization of tissues. As yet we know but in part, but we are accumulating a few postulates as the results of cultured discriminations as they are now being read from the work of nature through the microscope. And by this we may reasonably hope that the time is hastening when we will be able to throw aside the curtain of mystery and reveal the deductions. While I have referred to the more general manifestations of this disease, I have not noticed that there are many exciting causes, viz., mechanical irritations, dead pulps, alveolar abscess, crowded conditions of the teeth, accumulations of foreign substances, etc., etc. Not a few cases manifest a peculiar phase, noticed particularly associated with the exhibit of a recession of gum tissue and not any inflammatory action apparent. These are denominated atrophy of the gum. It is thought by many to be caused by friction of the brush. While this might, under some circumstances, facilitate the loss of tissue yet it is far from being the cause. This phase is seen at points where the brush would fail to have any such effect, viz., not only on the labial and buccal, but on the lingual and palatal surfaces of the teeth; and I would add that in many of these cases there is no perceptible presence of deposits of lime.

You will notice that I have, in passing, pointed out the manifestations of the disease in their mildest expressions. Starting out with the familiar adage that prevention is better than cure, it becomes of decided importance to emphasize familiarity with incipient stages, for if intelligence is active at this stage, we have under control the staying of its future destructiveness. The *serious* effects of this disease upon the general health are so well known to those who have familiarized themselves by an earnest and vig orous study of its workings, that it would be a crime to sit in silence and not proclaim the agonies associated. I am satisfied that large numbers are being cut off from their pilgrimage here prematurely, while thousands are dragging out a drooping existence of lassitude, depression and inanition directly and indirectly traceable to this disease. Perhaps I cannot do better than to state a case which will serve the purpose of demonstrating the many.

In the fall of 1878, Dr. Mason, Sr., Pres't. of the Long Island Medical College, called at my offce and consulted me about a patient of his who was in a wretched and rapid state of decline of health. He said he and his son had exhausted their remedies upon this patient, and he, having seen my articles published in Brochure, had become impressed that possibly this patient was a victim to the disease I had called his attention to. Several dentists had been consulted, but not with encouragement, excepting the extraction of the teeth. The patient came into my hands. She was about thirty-eight years of age, strong, nervous-bilious temperament, married. I found her with a dry, parched skin, feeble pulse, loss of appetite, depressed—sadly—sleepless, nausea on waking in the morning, and great loss of nerve energy. She had twenty-nine beautifully formed teeth, so loose that she had not been able to masticate with any power for a long time—some two years I think. From every socket was exuding a fetid discharge and very copious, so much so that she was obliged to place two large napkins under the side of her face to receive this flow at night while she slept. Now, this case did not prove to be one of suppurative pericementitis alone; it had involved the osseous formations, and that portion involving the sockets of the teeth was more or less destroyed. This case proved in treatment the necessity of surgical atttention in the direction claimed by Dr. Riggs, as I have described. It also proved that it had been developed altogether during the time she had been cognizant of it, but circumstances of such severity had fastened upon her and so checked the activities of her organization that it was left with an enfeebled power to cope with the disorder already present. The patient being so highly organized, her sufferings were of the acute order, and played great havoc among the distributions of the sensory nerves. The result of the treatment in this case, both surgical and constitutional, brought the patient again into the sphere of activity and usefulness. To use her own words, "she was as good as new." The exciting cause of the rapid decline of this patient was a *terrific mental grief.* I could detail numerous cases that have come under my notice during the last eight years, particularly where a variety of associate disorders had become complicated. I do not need to pursue the enumeration of these facts. You are familiar with many instances where the progress of disorders frequently reveal before unknown ones, resulting in prolonged distress, and not uncommonly, loss of life.

And now I do not think I need go further, for I do not doubt that the intelligent mind will grasp a proper measure of the truths to which I have called your attention, and which are readily demonstrated in the oral cavities of ninety per cent. of the people, in a greater or less degree of activity. I will not leave you with the impression that the specialty I represent is wholly alive, or in a large sense cognizant of the nature or of the destruction that is traveling madly over their every-day practice. The importance of surgical ability, as I said in my introductory, is becoming to be felt as the advanced step necessary to bring this branch to its needed elevation for usefulness. As yet dentistry, as practiced by the masses, can claim to be no more than Webster gives them credit for: "one who repairs teeth." To my understanding dentistry has a distinctively separate line from oral surgery, and will, I predict, in the near future be so estimated by intelligent people of discernment,

Gentlemen, it is from your ranks that much aid can come to assist those that are zealously devoting their energies to raise this special feature—oral surgery—to its sphere of greater usefulness in the alleviation of human suffering. And if you have been convinced, by what I have brought to your attention, of a conception of its importance, then I will have not spoken in vain.

The resume of this paper leads me to say this: that the revival of interest in this subject, by being brought up under a new feature, has proved aggressive, and by the controversy, interrogation by thought and action has given additional knowledge. It can no longer be viewed as a trival matter, for the fact is established that it is a specific disease, exhibiting specified manifestations and amenable to treatment, under the same limitations as all diseases; also, that trained perception and cultured discrimination, gained by concentrated investigations and practice, produce a grade of skill above that of the novice. Further, that the serious import of this subject to the public cannot be emphasized too strongly, for they cannot know too early that which is *first* our duty to become acquainted and impressed with, and in proportion as we come into possession of this knowledge, and are made conscious of its purpose in our hands, we will impress them by the alleviation of their sufferings. "He that hungers and thrists for knowledge, will, in the giving of it, unconsciously use it as a blessing and a joy to many."

---

